# Intracellular FGF1 protects cells from apoptosis through direct interaction with p53

**DOI:** 10.1007/s00018-023-04964-9

**Published:** 2023-10-02

**Authors:** Agata Lampart, Daniel Krowarsch, Martyna Biadun, Vigdis Sorensen, Jakub Szymczyk, Katarzyna Sluzalska, Antoni Wiedlocha, Jacek Otlewski, Malgorzata Zakrzewska

**Affiliations:** 1 Department of Protein Engineering, Faculty of Biotechnology, University of Wroclaw, Wroclaw, Poland; 2 Department of Protein Biotechnology, Faculty of Biotechnology, University of Wroclaw, Wroclaw, Poland; 3 Advanced Light Microscopy Core Facility, Dept. Core Facilities, Institute for Cancer Research, The Norwegian Radium Hospital, Oslo University Hospital, Montebello, Oslo Norway; 4 Centre for Cancer Cell Reprogramming, Institute of Clinical Medicine, Faculty of Medicine, University of Oslo, Montebello, Oslo Norway; 5 Department of Molecular Cell Biology, Institute for Cancer Research, Oslo University Hospital, Montebello, Oslo Norway

**Keywords:** Fibroblast growth factor 1 (FGF1), p53, Apoptosis, Protein–protein interaction, Intracellular function, Anti-apoptotic activity

## Abstract

Fibroblast growth factor 1 (FGF1) acts by activating specific tyrosine kinase receptors on the cell surface. In addition to this classical mode of action, FGF1 also exhibits intracellular activity. Recently, we found that FGF1 translocated into the cell interior exhibits anti-apoptotic activity independent of receptor activation and downstream signaling. Here, we show that expression of FGF1 increases the survival of cells treated with various apoptosis inducers, but only when wild-type p53 is present. The p53-negative cells were not protected by either ectopically expressed or translocated FGF1. We also confirmed the requirement of p53 for the anti-apoptotic intracellular activity of FGF1 by silencing p53, resulting in loss of the protective effect of FGF1. In contrast, in p53-negative cells, intracellular FGF1 regained its anti-apoptotic properties after transfection with wild-type p53. We also found that FGF1 directly interacts with p53 in cells and that the binding region is located in the DBD domain of p53. We therefore postulate that intracellular FGF1 protects cells from apoptosis by directly interacting with p53.

**Supplementary Information:**

The online version contains supplementary material available at 10.1007/s00018-023-04964-9.

## Introduction

As p53 protein plays a central role in cell cycle regulation and apoptosis, deregulation of its function occurs in most human cancers and several neurodegenerative diseases [1, 2]. Under physiological conditions, the amount of p53 protein in the cell is low, but in response to various stress stimuli, such as DNA damage, hypoxia, nutrient depletion, oxidative stress and nucleolar stress, p53 is activated and its levels increase rapidly [3]. The p53 protein acts within the cell nucleus as a transcription factor that activates the expression of many proteins, including p21, Bax, Noxa, PUMA, Apaf-1, Dram1, Sestrin1/2, taking part in a variety of processes critical for cell fate, such as cell cycle arrest, autophagy, apoptosis and metabolism regulation [4]. In addition to nuclear activity, p53 controls autophagy, centrosome duplication and apoptosis in the cytosol and mitochondria through transcription-independent mechanisms [5–7]. In addition to functioning as a tumor suppressor, p53 also modulates cellular senescence, playing important role in aging and the disposal of damaged post-mitotic cells [8–10], such as neurons affected in stroke or cardiac myocytes in an ischemic episode [11, 12].

Fibroblast growth factor 1 (FGF1) is a member of the FGF family, which consists of 22 members in humans. FGFs are versatile factors involved in proliferation, migration, differentiation and survival of different cell types [13]. The mechanism of action of FGFs depends on their binding to specific tyrosine kinase receptors (FGFR1-4), followed by their dimerization and activation. This leads to the recruitment of specific adaptor molecules and the initiation of a number of signaling cascades, including MAPKs, PI3K/Akt, PLCγ and STAT pathways [14]. Aberrations at different levels of the FGF/FGFR axis result in a wide variety of disorders, including developmental or metabolic diseases and various cancers [14, 15].

The two best-characterized members of the FGF family, FGF1 and FGF2, have a unique feature among other growth factors, namely the ability to translocate into the cytosol and nucleus upon binding to FGFR under stress conditions [16, 17]. For many years, the role of this process remained unknown, but our recent studies have uncovered the function of intracellular FGF1 and FGF2, showing that their translocation provides a specific intracrine signal, independent from the receptor activation, that protects the cells from apoptosis [18].

Here, we show that FGF1 interacts directly with p53 inside the cell and that this interaction is critical for the anti-apoptotic effect of intracellular FGF1. Expression of FGF1 or translocation of exogenously added FGF1 increases the survival of p53-positive cell lines exposed to various stress conditions. No protective effect of FGF1 is observed in p53-null cells or in p53-silenced cells. This indicates that, within the cell, FGF1 dictates cell fate by binding to p53.

## Materials and methods

### Antibodies and reagents

Stress inducers and inhibitors: PD173074, staurosporine, anisomycin, etoposide, brefeldin A, thapsigargin, doxorubicin and Fas ligand were from Sigma-Aldrich (MO, USA). Actinomycin D and camptothecin were from Santa Cruz Biotechnology (TX, USA). ABT 737, BTSA-1, BGJ398, ARQ087 were from Selleckchem (TX, USA), Apo2/TRAIL was from Merck (Darmstadt, Germany). The following primary antibodies were used: rabbit anti-PARP1/2 (9542), rabbit anti-BAX (D2E11) (5023), rabbit anti-phospho-p44/42 MAPK (ERK1/2) (Thr202/Tyr204) (9101) from Cell Signaling Technology (MA, USA), mouse anti-gamma tubulin (T6557) from Sigma-Aldrich, goat anti-FGF1 (sc-1884), mouse His-tag (H3) (sc-8036) and mouse anti-p53 DO-1 (sc-126) from Santa Cruz Biotechnology. Rabbit polyclonal antibodies against FGF1 (OPA1) were generated by Davids Biotechnologie GmbH (Regensburg, Germany) by immunizing rabbits with purified FGF1. The following HRP-conjugated secondary antibodies from Jackson Immuno Research Laboratories (PA, USA) were used: peroxidase affinipure goat anti-rabbit IgG (111-035-144) and anti-mouse IgG (115-035-003).

### Cell lines

The primary human foreskin fibroblast BJ cell line (CRL-2522), human osteosarcoma U2OS cell line (HTB-96), human breast cancer MCF7 cell line (HTB-22), human osteosarcoma G292 cell line, human prostate cancer PC3 cell line (CRL-1435) and human embryonic kidney HEK-293 cell line (CRL-1573) were from ATCC (VA, USA). U2OS cell lines stably transfected with wild type FGFR1 (U2OS_R1) were kindly provided by Dr. Ellen M. Haugsten from the Department of Molecular Cell Biology (Institute for Cancer Research, Oslo University Hospital) [19]. BJ, MCF7, G292 and U2OS cells were cultured in DMEM supplemented with 10% fetal bovine serum (FBS, Life Technologies, CA, USA). For U2OS_R1 cells, 0.5 mg/mL G418 sulfate (BioShop, Canada) was added to the culture medium. PC3 cells were grown in RPMI-1640 with 10% FBS. HEK 293 cells were cultured in MEM medium supplemented with 10% FBS. All culture media were supplemented with antibiotics (100 U/mL penicillin and 100 µg/mL streptomycin) from Life Technologies (CA, USA). Cells were cultured in a 5% CO_2_ atmosphere at 37 °C. All cell lines were routinely tested for mycoplasma contamination.

### Vectors

The pET-3c vectors (Novagen, WI, USA) with DNA fragments encoding FGF1 (Ala- FGF1^22−155 ^or SBP-tagged Ala-FGF1^22–155^) were used to express FGF1 in the bacterial system [20]. For cell transfection experiments, sequences encoding N-terminal myc- or SBP-tagged FGF1 were cloned into the pcDNA3.1 vector (Invitrogen, CA, USA). Constructs encoding the his-tagged full-length p53 (p53^1−393^) and the p53 DNA-binding domain (p53_DBD: p53^94−312^) in the pET15b vector for bacterial expression were obtained from Cheryl Arrowsmith via Addgene (plasmid #24859 and #24866) [21]. The wild type hBax C3-EGFP (Bax-EGFP) construct was provided by Richard Youle through Addgene (plasmid #19741) [22]. The pcDNA-DEST47 vector with a DNA fragment encoding the full-length wild type p53 was used to transfect p53-negative cell lines.

### Protein expression and purification

FGF1 proteins were expressed in *E. coli* strain BL21(DE3)pLyS (Invitrogen) and purified using Heparin-Sepharose Fast Flow resin (GE Healthcare, UK), as described previously. The native conformation of purified proteins was confirmed by circular dichroism (J-715 spectropolarimeter, Jasco, MD, USA) and fluorescence (FP-750 spectrofluorimeter, Jasco, MD, USA) [23]. His-tagged p53 full-length and p53_DBD were produced in *E.Coli* Arctic Express strain (Agilent Technologies, CA, USA) at 16 ℃, in LB medium supplemented with 30 nM ZnCl_2_. Protein expression was induced at OD_600_ = 1 by adding of IPTG to a final concentration of 0.5 mM and continued for 16 h. Bacteria were then harvested by centrifugation and resuspended in buffer containing 20 mM HEPES, 150 mM NaCl, 20 mM imidazole, 30 nM ZnCl_2_, pH 7.8 and 500 U/L of Pierce Universal Nuclease (Thermo Fischer Scientific, MA, USA), followed by sonication. A Ni Sepharose High Performance column (GE Healthcare, UK) was equilibrated with binding buffer (20 mM HEPES, 300 mM NaCl, 20 mM imidazole, 0.15% Triton X-100, 30 nM ZnCl_2_, pH 7.8), followed by application of soluble His-tagged p53 full-length or p53_DBD bacterial extracts and incubation over-night at 4 ℃. The column was then washed with ZnCl_2_ and Triton X-100-free binding buffer (20 mM HEPES, 300 mM NaCl, 20 mM imidazole, pH 7.8) until A_280_ was stabilized. Proteins were eluted with 20 mM HEPES, 100 mM NaCl, 300 mM imidazole, pH 7.8. His-tagged p53_DBD fractions were then subjected to dialysis against 20 mM HEPES, 100 mM NaCl, pH 7.8. An additional purification step was used for full-length His-p53. His-p53 samples diluted with 20 mM HEPES, 100 mM NaCl, pH 7.8 were applied to a HiTrap DEAE Sepharose Fast Flow column (GE Healthcare), washed with the same buffer and eluted in a continuous 0–100% gradient with 20 mM HEPES, 500 mM NaCl, pH 7.8. The purity and molecular identity of the proteins obtained were verified by SDS-PAGE and mass spectrometry on a 4800 Plus MALDI TOF/TOF instrument (Applied Biosystems, UK).

### Transfection and siRNAs

Transient expression of myc-FGF1, SBP-FGF1, Bax-EGFP and p53 was performed by transfecting selected cell lines with plasmid DNA using FuGene HD transfection reagent (Promega, WI, USA) or Lipofectamine LTX & Plus Reagent (Invitrogen) according to the manufacturer’s protocol. Cells were seeded at 60–80% confluence the day before transfection. Experiments were performed 48 h post transfection. FGF1 expression levels were analyzed by immunoblotting using an anti-FGF1 antibody. For stable transfection, cells were cultured in selection medium containing 0.5 mg/mL G418 sulfate. Three clones from each selection were chosen for further analysis. siRNA targeting p53 (HSS186390) was purchased from Thermo Fisher Scientific and scrambled control siRNA (D-001810-01-05) was obtained from Dharmacon Horizon Discovery (CO, USA). For the siRNA transfection experiments, U2OS cells (stably transfected with the empty pcDNA3.1 vector or myc-FGF1 construct) or U2OS_R1 cells were seeded at 60% confluence and transfected with 40 nM siRNA targeting p53 and control non-targeting siRNA, using DharmaFECT 1 Transfection Reagent (Dharmacon Horizon Discovery) according to the procedure provided by the company. Eight hours after transfection, 10% FBS was added to the cells, and the cells were cultured for a further 24 h. Then, cells were seeded on 6- or 96-well plates for further experiments.

### Annexin V assay

U2OS cells stably transfected with FGF1 or control vector were treated with 10 μM anisomycin or 5 μM actinomycin D for 24 h to induce apoptosis. All experiments were conducted in the presence of a potent FGFR kinase inhibitor, 100 nM PD173074, to ensure that observed anti-apoptotic effects originated exclusively from intracellular FGF1. Cells were then detached by incubation with trypsin–EDTA solution (Life Technologies, CA, USA), harvested and stained with annexin V and 7AAD using Muse Annexin V and Dead Cell Assay Kit (Merck), as previously described [18]. Samples were subjected to flow cytometry using the Muse Cell Analyzer (Merck), and the resulting data were analyzed using Muse 1.3.1 Analysis software (Merck). All experiments were performed three times (n = 3) with three replicates in each experiment.

### Cell viability assay

Cell viability measurements were performed using Presto Blue reagent (Thermo Fisher Scientific) according to the manufacturer’s protocol 24 h after treating cells with apoptosis inducers in the presence or absence of FGFR kinase inhibitors, 100 nM PD173074, 10 nM BGJ398 or 1 μM ARQ087. Cell viability was then normalized to untreated control cells. All experiments were performed three times (n = 3) with three replicates in each experiment.

### Caspase-3/7 activity

Cells stably or transiently transfected with FGF1 and control cells were subjected to induction of apoptosis by starvation or treatment with stress inducers for 24 h in the presence or absence of FGFR kinase inhibitors, 100 nM PD173074, 10 nM BGJ398 or 1 μM ARQ087. Cell viability and caspase-3/7 activity were then measured using the ApoLive-Glo Multiplex Assay (Promega), according to the manufacturer's protocol. The ratio of caspase-3/7 activity and cell viability was then normalized against control cells not treated with stress inducers and denoted as relative caspase-3/7 activity. U2OS_R1 and G292 cells were subjected to induction of apoptosis by starvation for 24 h and then treated with recombinant FGF1, in the presence of 100 nM PD173074 and 10 U/mL heparin for an additional 16 h. Subsequently, cell viability and caspase-3/7 activity were measured using the ApoLive-Glo Multiplex Assay. In this case, the ratio of caspase-3/7 activity and cell viability was normalized to the negative control, starved cells treated with PD173074 and heparin alone. All experiments were performed three times (n = 3) with three replicates in each experiment.

### Cell fractionation

Serum-starved cells were incubated with FGF1 for 6 h and then were washed with high salt/low-pH-buffer (HSLP, 2 M NaCl, 20 mM sodium acetate, pH 4.0) to remove surface-bound FGF1 and in PBS before fractionation. In order to fractionate the cells into membrane, cytosolic and nuclear fractions, the digitonin fractionation method was used as previously described [24]. Cells were permeabilized with 20 µg/ml digitonin in PBS and incubated at room temperature for 5 min and on ice for an additional 30 min to allow cytosol diffusion into the buffer. The buffer was collected and labelled as the cytosolic fraction. The remaining fraction of cells were lysed in lysis buffer (50 mM Tris, 150 mM NaCl, 0.1% Triton X-100, pH 7.4) supplemented with a protease inhibitor cocktail (Merck). Cell lysates were centrifuged at 15 000 × g and the soluble fraction was collected as the membrane fraction. The insoluble fraction was labelled as the nuclear fraction. The nuclear fraction was washed in lysis buffer to get rid of cytoplasmic debris and disrupted by sonication. FGF1 was extracted from the isolated subcellular fractions by Heparin-Sepharose pull-down and analyzed by SDS-PAGE and immunoblotting.

### In situ proximity ligation assay (PLA)

PLA experiments were performed using Duolink reagents according to the manufacturer’s protocol (Sigma-Aldrich). U2OS cells stably transfected with myc-FGF1_pcDNA3.1 or control (empty) pcDNA3.1 vector were seeded onto coverslips at 60% confluence 24 h before the PLA procedure. Cells were fixed with formaldehyde and permeabilized with PBS containing 1% Triton X-100. The following antibodies were used to detect p53-FGF1 complexes: 1:500 mouse anti-p53 DO1 and 1:500 goat anti-FGF1. After 2-h incubation at room temperature with the primary antibodies, PLA probes anti-rabbit PLUS and anti-mouse MINUS were applied. Amplification was performed overnight at 37 ℃ and the remaining steps were performed according to the manufacturer’s protocol. For each antibody, a negative control experiment was performed in which the antibody was incubated with PLA probes. Cell nuclei were counterstained with DAPI (Sigma-Aldrich). Forty Z-stacks of different parts of each sample were taken using an LSM 710 Zeiss Confocal Microscope with a 63 × objective. 2D images were then created and PLA signals were quantified.

### Pull-down assays

Untransfected U2OS cells or U2OS cells transfected with SBP-FGF_pcDNA3.1 or pcDNA3.1 control vector were lysed in lysis buffer (50 mM Tris–HCl, 150 mM NaCl, 1% Triton, pH 7.4) supplemented with protease inhibitor cocktail and sonicated three times for 5 s. Cellular debris was removed by centrifugation and protein concentration was measured using the Bradford assay to ensure equal sample loading. Cleared lysates were incubated with 30 µl of Pierce Streptavidin-Agarose resin (Thermo Fisher Scientific) for 2 h at 4 ℃ with shaking. In all cases, the resins were washed three times in lysis buffer before elution of the protein complexes by boiling for 10 min in SDS sample buffer. A similar procedure was applied to study the interaction of recombinant SBP-FGF1 with p53 from U2OS cell lysate and to analyze the interaction of recombinant FGF1 with recombinant his-tagged p53 and p53_DBD.

### SPR measurements

Interactions between recombinant proteins were measured by surface plasmon resonance (SPR) using a Biacore 3000 instrument. (GE Healthcare) at 25 ℃. The recombinant human full-length p53 and its DNA-binding domain (p53_DBD) dissolved in 10 mM sodium acetate, pH 5.0 were immobilized on CM5 sensor chip surface (GE Healthcare) at about 1000 RU and 800 RU, respectively, using an amine coupling protocol. To determine interaction parameters between FGF1 and p53 or p53_DBD, measurements were performed in 10 mM HEPES, 150 mM NaCl, 0.05% Tween 20, 0.1% BSA, 0.02% NaN_3_, pH 7.4. Recombinant FGF1 protein at the concentrations ranging from 16 to 2048 nM was injected at a flow of 30 μl/min. The association and disassociation phases were monitored for 4 min and 5 min, respectively. After each measurement the sensor was regenerated with 2.5 M NaCl and 10 mM NaOH solution. The data were analyzed using the BIAevaluation 4.1 software (GE Healthcare). Equilibrium dissociation constant (K_D_) was calculated from fitted saturation binding curve [25]. Response values from the last 10 s of the association phase were averaged and used to determine the K_D_.

### Statistical analysis

A paired one-tailed Student's t-test was used for statistical analysis using SigmaPlot software (Systat Software).

## Results

### FGF1 expression increases the resistance of U2OS cells to apoptosis induced by the intrinsic pathway

**Fig. 1:**
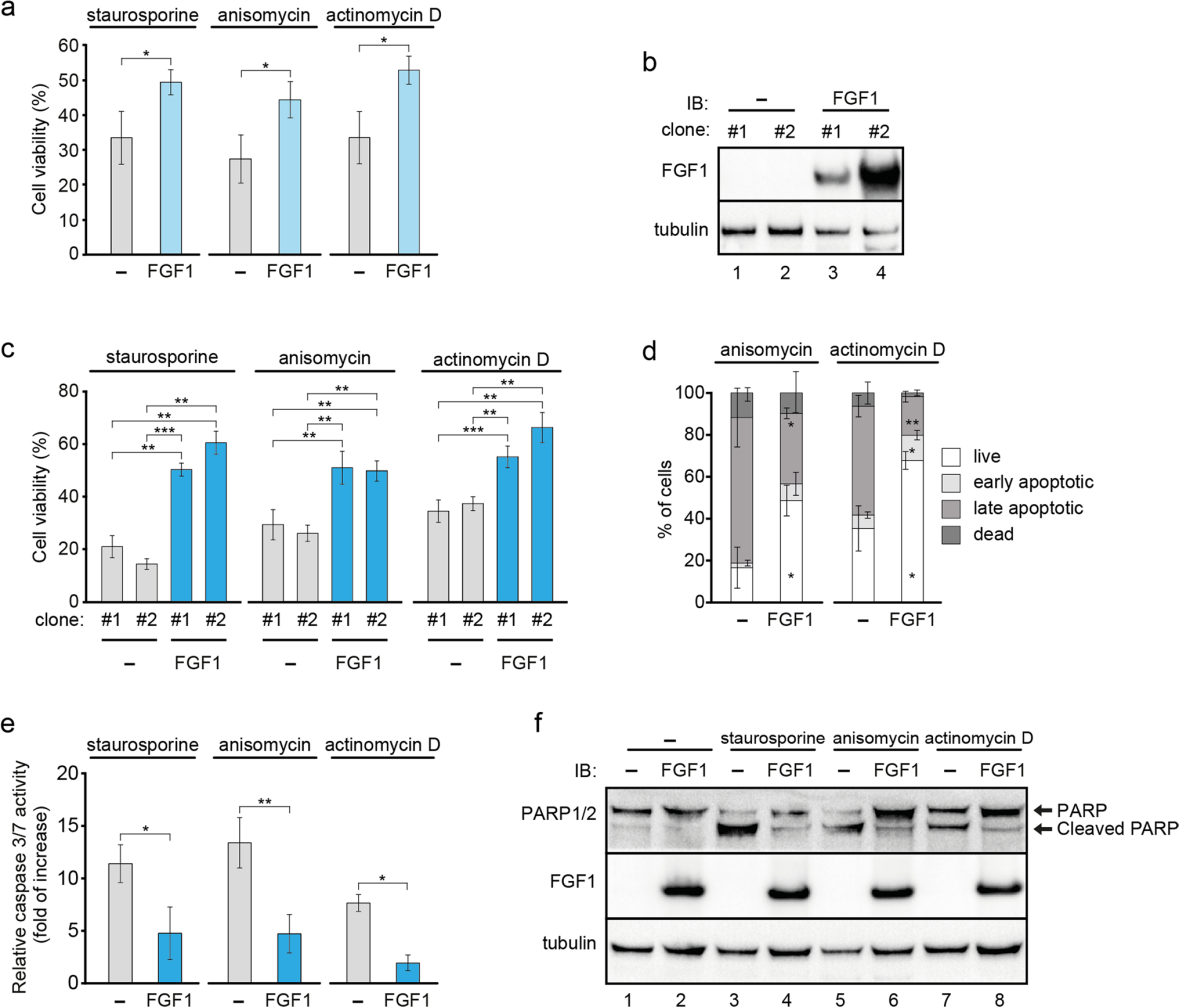
FGF1 expression provides anti-apoptotic protection of U2OS cells against various apoptotic factors. **a** Viability of U2OS cells transiently transfected with myc-FGF1_pcDNA3.1 (FGF1) or control pcDNA3.1 (–) vectors and treated with 1 μM staurosporine, 10 μM anisomycin or 5 μM actinomycin D in the presence of FGFR kinase inhibitor (100 nM PD173074) for 24 h. Cell viability was measured using Presto Blue reagent. Graphs show means ± SD from three independent experiments. Statistical significance: *p < 0.05. **b** Western blot analysis of FGF1 protein levels in two independent clones of U2OS cells stably transfected with myc-FGF1_pcDNA3.1 (FGF1) or empty pcDNA3.1 (–) vectors. Anti-gamma-tubulin antibody served as a loading control. **c** Viability of U2OS cells stably transfected with myc-FGF1_pcDNA3.1 (FGF1) or empty pcDNA3.1 (–) vectors (two clones each, #1, #2) treated with 1 μM staurosporine, 10 μM anisomycin or 5 μM actinomycin D in the presence of FGFR kinase inhibitor (100 nM PD173074). Cell viability was measured using Presto Blue reagent 24 h after treatment. Graphs show means ± SD from three independent experiments. Statistical significance: **p < 0.01, ***p < 0.001. **d** Apoptosis progression was monitored in U2OS cells stably transfected with myc-FGF1_pcDNA3.1 (FGF1) or empty pcDNA3.1 (–) vectors after 24-h treatment with 10 μM anisomycin or 5 μM actinomycin D in the presence of 100 nM PD173074 using Muse Annexin V and Dead Cell Assay Kit. Data are presented as percentage of live, early apoptotic, late apoptotic and dead cells. Graphs show means ± SD from three independent experiments. Statistical significance: *p < 0.05, **p < 0.01. **e** Relative caspase-3/7 activity measured in U2OS cells stably transfected with myc-FGF1_pcDNA3.1 (FGF1) or empty pcDNA3.1 (–) vectors after 24-h treatment with 1 μM staurosporine, 10 μM anisomycin or 5 μM actinomycin D in the presence of 100 nM PD173074 using ApoLive-Glo Multiplex Assay. Graphs show means ± SD of three independent experiments. Statistical significance: *p < 0.05, **p < 0.01. **f** PARP-1/2 cleavage determined in U2OS cells stably transfected with myc-FGF1_pcDNA3.1 (FGF1) or empty pcDNA3.1 (–) vectors after 24 h treatment with 1 μM staurosporine, 10 μM anisomycin or 5 μM actinomycin D in the presence of 100 nM PD173074. Anti-gamma-tubulin antibody served as a loading control

We have previously shown that U2OS cells transiently transfected with FGF1 or FGF2 treated with staurosporine, a well-known inducer of apoptosis in a wide range of cell lines [26], were more resistant to apoptosis than untransfected cells as detected by analysis of caspase 3/7 activity [18]. Here, we confirmed this observation using two other inducers of apoptosis, anisomycin, which blocks protein translation and activates SAPK/JNK and p38 MAPK cascades [27], and actinomycin D, which interacts with DNA to inhibit its transcription and induce nucleolar stress and apoptosis [28, 29]. Cells transiently transfected with myc-FGF1_pcDNA3.1 or empty pcDNA3.1 vector (control) were treated with 1 μM staurosporine, 10 μM anisomycin or 5 μM actinomycin D for 24 h and then cell viability was measured using Presto Blue reagent. The level of FGF receptors in U2OS cells is very low (undetectable by western Blot analysis [18]), nevertheless, we performed experiments in the presence of a specific FGFR kinase inhibitor, PD173074, to ensure that the observed activity was not due to residual FGFR activation upon potential FGF1 secretion or release. Consistent with previously published data for all apoptosis-induced agents, we observed an approximately one-and-a-half-fold increase in the viability of cells expressing the FGF1 construct compared to cells transfected with the empty vector (Fig. 1a). To verify these results, we additionally used two other chemically unrelated, highly specific FGFR kinase inhibitors, BGJ398 and ARQ087 (Fig. S1a). Again, we observed a statistically significant anti-apoptotic effect of transient FGF1 expression. Using western blot analysis of MAPK activation, we confirmed that FGFR signaling in the presence of staurosporine and PD173074 or BGJ398 is completely inhibited up to 24 h (Fig. S1b).We then performed the above experiments using U2OS cells stably transfected with myc-FGF1_pcDNA3.1 or empty pcDNA3.1 vectors. FGF1 levels in selected clones were compared by western blot analysis (Fig. 1b). Using two isolated clones, we tested protective properties of FGF1 in response to 1 μM staurosporine, 10 μM anisomycin or 5 μM actinomycin D. We analyzed cell viability after 24 h of treatment with stress inducers and observed a protective effect of FGF1 expression in each case (Fig. S2a). Both FGF1_U2OS clones tested showed significantly higher viability than U2OS clones transfected with empty vector. In the same way, we performed the experiments in the presence of a specific FGFR kinase inhibitor, PD173074 (Fig. 1c). The results were very similar to those obtained in the absence of the inhibitor, but to fully ensure that the observed effects were due only to intracellular activity of FGF1 and not due to residual FGFR activation, potentially occurring as a result of secretion or release of FGF1 from the disrupted cells, PD173074 was present in all further experiments. The protective effect of FGF1 protein was even more pronounced 48 h after induction of apoptosis with 5 μM actinomycin D (Fig. S2b). Since both clones of the U2OS and FGF1_U2OS behave virtually identically, we decided to use only one clone of each cell line (clones #1) in the remainder of the study. In the case of FGF1_U2OS cells, we confirmed, by western blot analysis of MAPK activation, that in the presence of all apoptosis inducers used (staurosporine, anisomycin and actinomycin D) PD173074 completely blocks FGFR signaling up to 24 h (Fig. S2c). We also verified the cell viability results in the presence of two other FGFR kinase inhibitors, BGJ398 and ARQ087 (Fig. S2d). Apoptosis progression in U2OS cells was also monitored by flow cytometry using the Muse Annexin V and Dead Cell Assay Kit (Merck). We analyzed the state of the cells upon 24-h treatment with 10 μM anisomycin or 5 μM actinomycin D. In agreement with the result of the cell viability experiment, FGF1_U2OS cell line treated with both apoptosis inducers showed a significantly higher percentage of live cells than control U2OS cell line (Fig. 1d). To further investigate the effect of intracellular FGF1 on apoptosis, we analyzed changes in caspase 3/7 activity and PARP processing. For all stress inducers used, FGF1_U2OS cells showed significantly reduced caspase activity (Fig. 1e, Fig. S2e) and PARP cleavage (Fig. 1f) compared to control U2OS cells in the presence of FGFR kinase inhibitors, confirming the anti-apoptotic effect of intracellular FGF1.

**Fig. 2:**
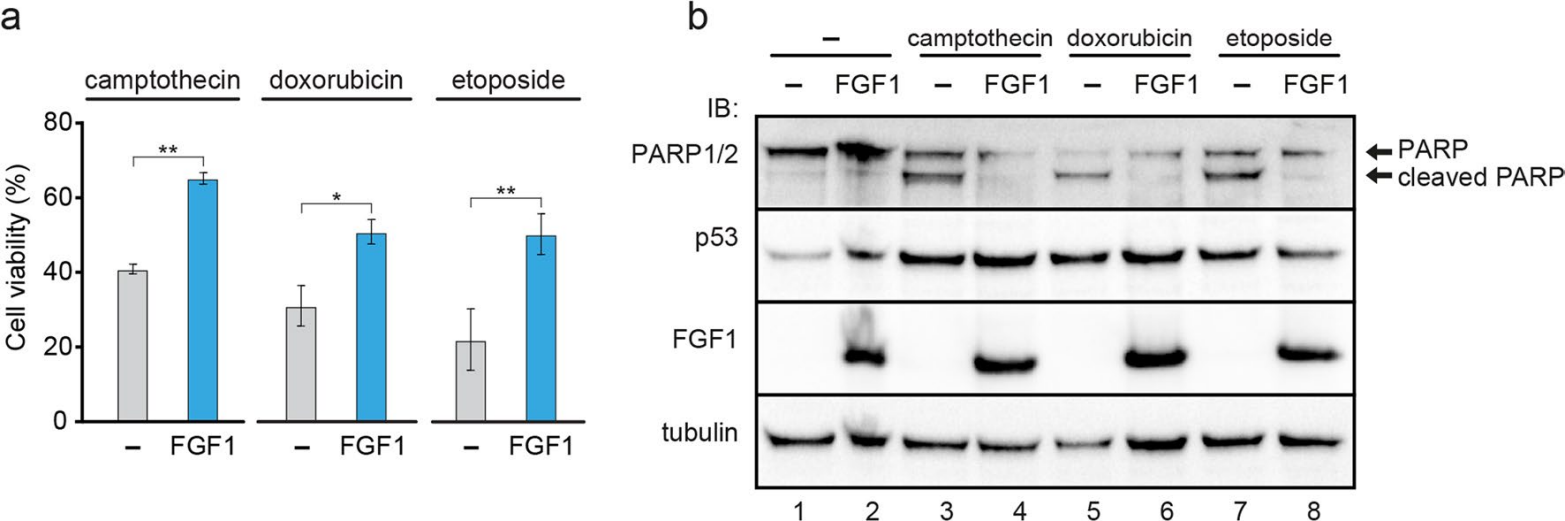
Intracellular FGF1 protects U2OS cells against p53-mediated apoptosis. **a** Viability of U2OS cells stably transfected with myc-FGF1_pcDNA3.1 (FGF1) or empty pcDNA3.1 (–) vectors treated with 5 μM camptothecin, 10 μM doxorubicin or 50 μg/mL etoposide in the presence of FGFR kinase inhibitor (100 nM PD173074). Cell viability was measured using Presto Blue reagent 24 h after treatment. Graphs show means ± SD from three independent experiments. Statistical significance: *p < 0.05, **p < 0.01. **b** PARP-1/2 cleavage and p53 protein levels in U2OS cells stably transfected with myc-FGF1_pcDNA3.1 (FGF1) or empty pcDNA3.1 (–) vectors after 24-h treatment with 5 μM campthotecin, 10 μM doxorubicin or 50 μg/mL etoposide in the presence of 100 nM PD173074. Anti-gamma-tubulin antibody served as a loading control

We then used several p53 activators as additional, more specific, stimulators of the intrinsic apoptosis pathway: 5 μM camptothecin [30], 50 μg/mL etoposide [31] and 10 μM doxorubicin [32] for 24-h incubation with FGF1_U2OS and control U2OS cells. FGF1-expressing U2OS cells showed significantly higher viability after treatment with all tested inducers than non-FGF1-transfected cells, despite effective blockade of FGFR signaling by specific FGFR kinase inhibitors (Fig. 2a, Fig. S3a, Fig. S3b). Furthermore, western blot analysis showed that FGF1 effectively inhibits PARP cleavage, but does not affect p53 levels (Fig. 2b). With etoposide treatment, we also verified caspase 3/7 activity, which was significantly reduced in cells expressing FGF1 (Fig. S3c). These results confirm that intracellular FGF1 inhibits p53-dependent apoptosis induced via the intrinsic pathway.

### FGF1 does not protect cells from apoptosis induced via the extrinsic pathway or ER-stress

**Fig. 3:**
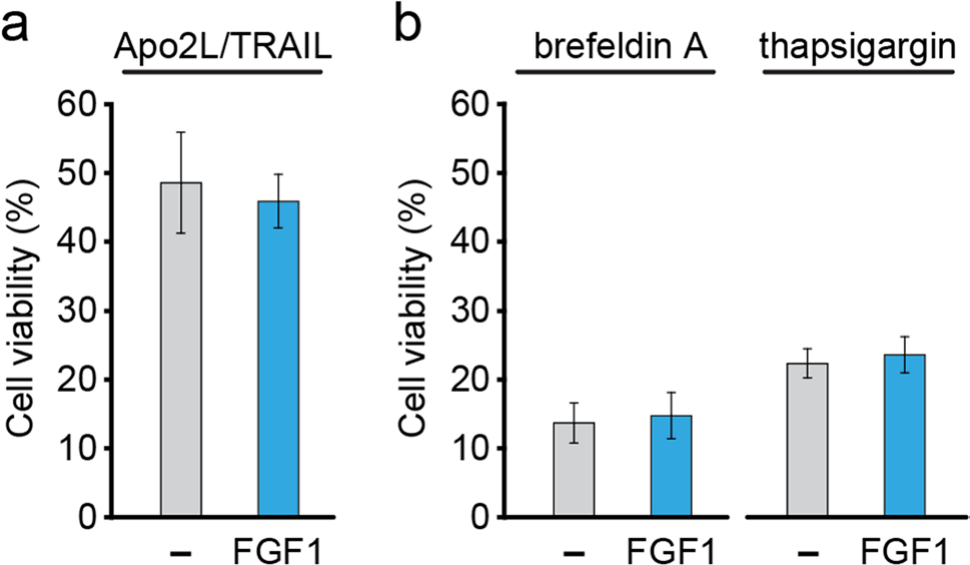
Intracellular FGF1 does not protect U2OS cells against apoptosis induced by extrinsic pathway or ER stress. **a** Viability of U2OS cells stably transfected with myc-FGF1_pcDNA3.1 (FGF1) or empty pcDNA3.1 (–) vectors treated with 250 ng/mL Apo2 ligand (Apo2L) in the presence of FGFR kinase inhibitor (100 nM PD173074). **b** Viability of U2OS cells stably transfected with myc-FGF1_pcDNA3.1 (FGF1) or empty pcDNA3.1 (–) vectors treated with ER stress inducing agents: 5 μM brefeldin A or 1 μM thapsigargin in the presence of 100 nM PD173074. Cell viability was measured using Presto Blue reagent 24 h after treatment. Graphs show means ± SD from three independent experiments. No statistical significance

To better characterize the intracellular action of FGF1, we verified its activity after inducing apoptosis via the extrinsic pathway. For this purpose, we used Apo2 ligand/TRAIL, which induces caspase-8 activation upon binding to death receptors on the cell surface [33]. U2OS cells expressing FGF1 or control cells were treated with 250 ng/mL of TRAIL for 24 h. We observed no differences in viability between FGF1_U2OS and control U2OS cells, suggesting that intracellular FGF1 does not protect cells from apoptosis via the extrinsic pathway (Fig. 3a). The same results were obtained for the Fas ligand (300 ng/mL), but in this case the induction of apoptosis was less effective (Fig. S4).

We then induced endoplasmic reticulum (ER) stress using brefeldin A and thapsigargin. Brefeldin A blocks protein transport to the Golgi apparatus leading to the accumulation of secretory proteins within the ER, thus generating ER stress [34]. Thapsigargin is an inhibitor of sarco/endoplasmic reticulum Ca^2+^ ATPase (SERCA), raising cytosolic calcium concentration while Ca^2+^ stores in the ER are depleted. Decreased calcium levels in the ER lead to ER stress and activation of the unfolded protein response [35, 36]. FGF1_U2OS and control U2OS cells were treated with 5 μg/mL brefeldin A or 1 μg/mL thapsigargin for 24 h to assess cell viability. We observed a lack of protective effect of intracellular FGF1, as the viability of both FGF1_U2OS and control cells was very similar after treatment with brefeldin or thapsigargin (Fig. 3b).

### FGF1 acts upstream of mitochondrial membrane permeabilization

To gain more insight into the mechanism of the protective effect of intracellular FGF1, we employed two other specific inducers of apoptosis, ABT-737, an inhibitor of Bcl-2 and Bcl-XL, and BTSA1, a Bax/Bak activator, which induce mitochondrial outer membrane permeabilization (MOMP). ABT-737 mimicks the BH3-peptide and binds to Bcl-2 and Bcl-XL, blocking their anti-apoptotic function [37], while BTSA1 is a small molecule that interacts with the N-terminus of Bax/Bak, leading to its activation and insertion into the outer mitochondrial membrane [38]. Both compounds induce apoptosis by permeabilizing mitochondria through either inhibition of anti-apoptotic Bcl-2 proteins or activation of pro-apoptotic Bax/Bak.

**Fig. 4:**
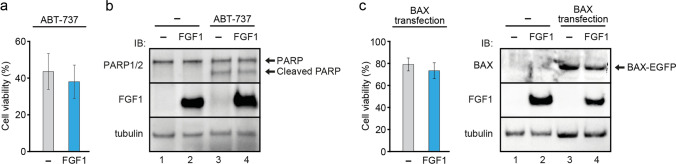
FGF1 is unable to inhibit apoptosis induced at the mitochondrial outer membrane permeabilization (MOMP) stage. **a** Viability of U2OS cells stably transfected with myc-FGF1_pcDNA3.1 (FGF1) or empty pcDNA3.1 (–) vectors treated with 10 μM ABT 737 in the presence of FGFR kinase inhibitor (100 nM PD173074). Cell viability was measured using Presto Blue reagent 24 h after treatment. The graph shows means ± SD from three independent experiments. No statistical significance. **b** PARP-1/2 cleavage determined in U2OS cells stably transfected with myc-FGF1_pcDNA3.1 (FGF1) or empty pcDNA3.1 (–) vectors after 24-h treatment with 10 μM ABT 737 in the presence of 100 nM PD173074. Anti-gamma-tubulin antibody served as a loading control. **c** U2OS cells stably transfected with myc-FGF1_pcDNA3.1 (FGF1) or empty pcDNA3.1 (–) vectors were transiently transfected with Bax-EGFP construct. Cell viability was measured 24 h after transfection using Presto Blue reagent. The graph shows means ± SD from three independent experiments. No statistical significance. The efficiency of Bax transfection was verified using an anti-Bax antibody. Anti-gamma-tubulin antibody served as a loading control

U2OS cells with or without FGF1 expression were treated with 10 μM ABT-737 or 30 μM BTSA1 for 24 h, and cell viability was then determined. We did not observe any protective effect of intracellular FGF1 upon ABT-737 treatment, as FGF1_U2OS cells did not show increased viability compared to control U2OS cells (43.7% *vs* 38.1%, Fig. 4a). Similarly, when analyzing PARP cleavage, we also found no difference in apoptotic response to ABT-737, regardless of FGF1 expression (Fig. 4b). Both cell lines appeared more resistant to BTSA1 treatment than to ABT-737, but again, we did not observe a protective effect of FGF1 on either viability or PARP processing (Fig. S5). We also induced apoptosis by directly increasing Bax protein level, transiently transfecting FGF1_U2OS and control U2OS cells with Bax-EGFP [22]. 24 h after transfection, both lines expressing high levels of Bax-EGFP showed reduced viability at similar level (Fig. 4b). These data suggest that intracellular FGF1 protects U2OS cells against apoptosis, acting before mitochondrial outer membrane permeabilization (MOMP) occurs.

### Transient FGF1 overexpression protects p53-positive but not p53-negative cells from apoptosis

**Fig. 5:**
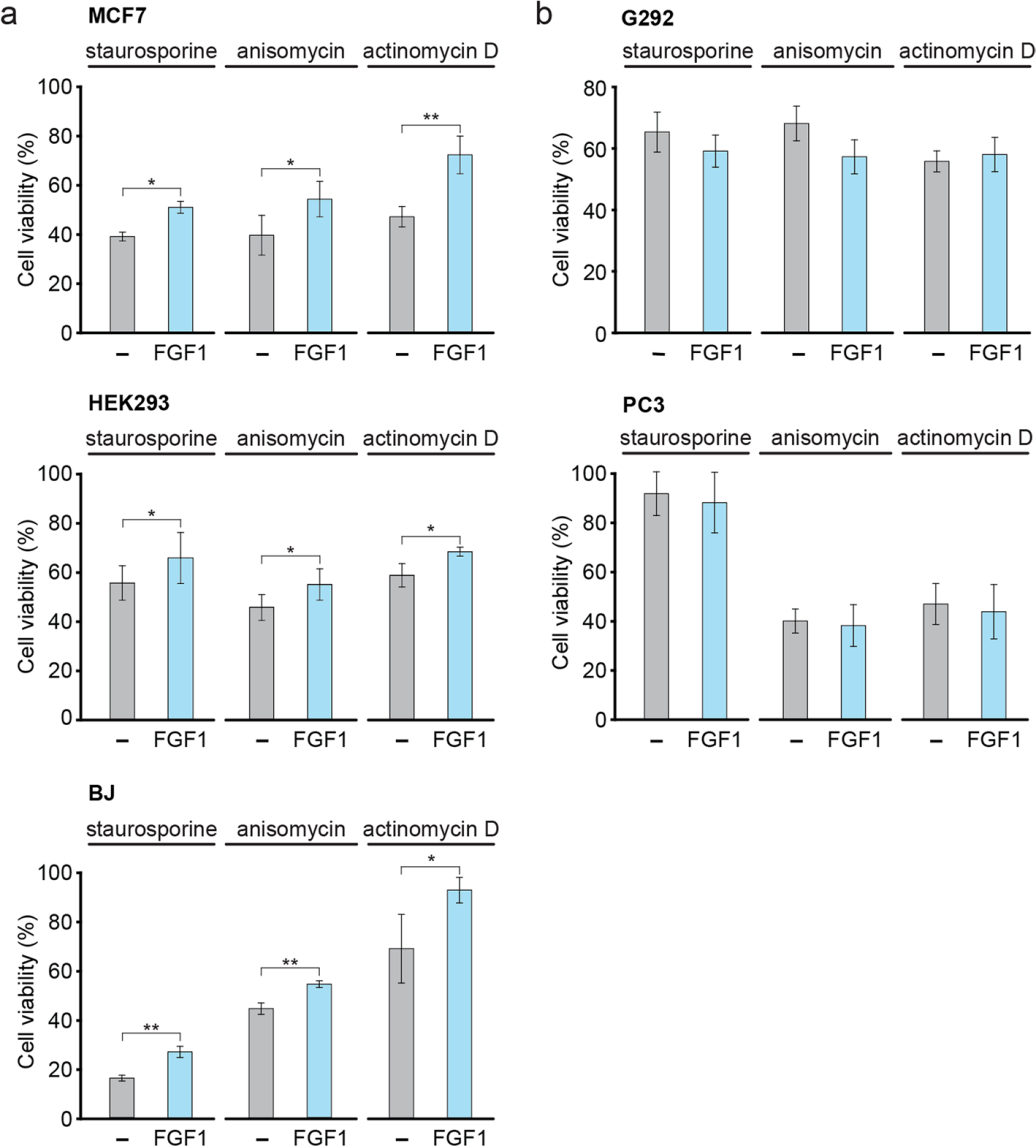
Transiently expressed FGF1 protects cell against apoptosis only in p53-positive cells. **a**, **b** MCF7, BJ and HEK 293 cell lines expressing WT p53 **(a)** and p53-negative cell lines G292 and PC3 **b** were transiently transfected with myc-FGF1_pcDNA3.1 or control pcDNA3.1 vectors. 48 h after transfection, cells were treated with treated with 1 μM staurosporine, 10 μM anisomycin or 5 μM actinomycin D in the presence of FGFR kinase inhibitor (100 nM PD173074) for 24 h. Cell viability was measured using Presto Blue reagent. Graphs show means ± SD from three independent experiments. Statistical significance: *p < 0.05, **p < 0.01

To test the anti-apoptotic activity of intracellular FGF1 in p53-positive cell lines other than U2OS, we used MCF7 (breast adenocarcinoma), BJ (normal fibroblasts) and HEK293 (embryonic human kidney) cells. We transiently transfected these cell lines with the FGF1_pcDNA3.1 vector or the empty pcDNA3.1 control vector (Fig. S5). 48 h after transfection, cells were treated with 1 μM staurosporine, 10 μM anisomycin or 5 μM actinomycin D to induce apoptosis and cell viability was determined 24 h later. In all cases, FGF1-transfected cells exhibited higher viability than control cells (transfected with empty pcDNA3.1). (Fig. 5a). We then performed similar experiments using two different p53-negative cell lines. We transiently transfected G292 (osteosarcoma) and PC3 (prostate adenocarcinoma) cells with the FGF1_pcDNA3.1 vector or the empty pcDNA3.1 control vector (Fig. S6) and performed apoptosis experiments using the same stress inducers as in the experiments with p53-positive cell lines. We observed no protective effect of FGF1 in G292 and PC3 cells treated with staurosporine, anisomycin or actinomycin D (Fig. 5b). These results suggest that the intracellular anti-apoptotic activity of FGF1 requires the presence of p53.

### Translocated FGF1 protects p53-positive, but not p53-negative osteosarcoma cells from serum starvation-induced apoptosis

**Fig. 6:**
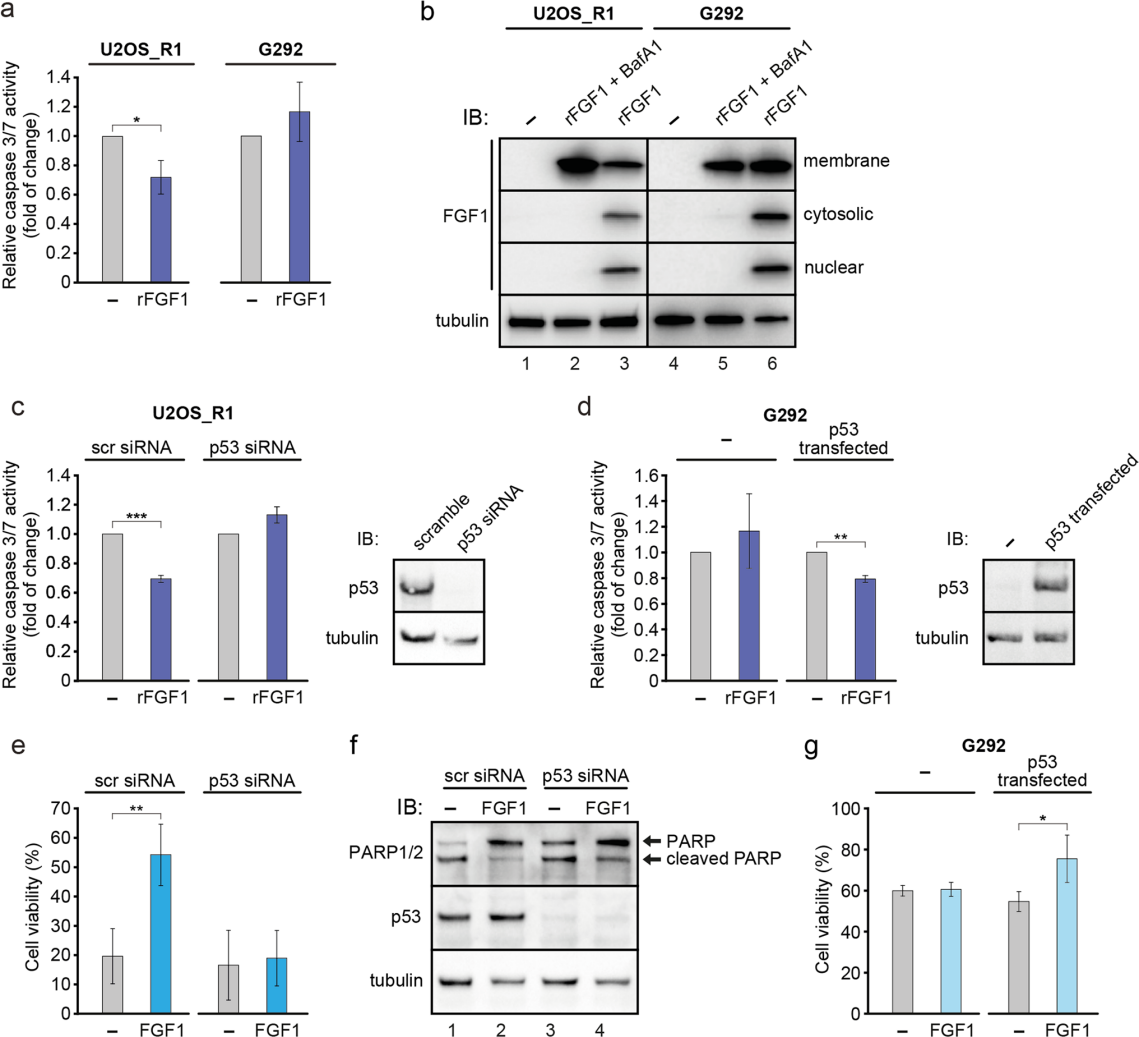
p53 is required for the anti-apoptotic activity of intracellular FGF1. **a** Translocated FGF1 protects p53-positive but not p53-negative osteosarcoma cells from serum starvation-induced apoptosis. Relative caspase-3/7 activity measured using ApoLive-Glo Multiplex Assay in serum-starved U2OS cells stably transfected with FGFR1_pcDNA3.1 vector (U2OS_R1) and G292 cells stimulated with 200 ng/mL FGF1 in the presence of 100 nM PD173074 and 10 U/mL heparin for 16 h. Graphs show means ± SD of three independent experiments. Statistical significance: *p < 0.05, **p < 0.01. **b** Translocation of 200 ng/ml recombinant FGF1 (rFGF1) into the cytosol and nucleus of serum-starved U2OSR1 and G292 cells in the presence of 10 U/mL heparin and 100 nM PD173074 after 6 h. 10 nM Bafilomycin A1 (BafA1), an inhibitor of FGF1 translocation, was used as in a negative control. Cells were fractionated into membrane (M), cytosolic (C) and nuclear (N) fractions. rFGF1 was extracted from each fraction by adsorption on Heparin-Sepharose resin and analyzed by SDS-PAGE and western blotting with anti-FGF1 antibody. To ensure equal loading, total lysates were also analyzed using an anti-gamma-tubulin antibody. **c** Effect of p53 knockdown in U2OS_R1 cells. Serum-starved U2OS_R1 cells transfected with p53 siRNA or a non-targeting siRNA control (scr) were stimulated with 200 ng/mL rFGF1 in the presence of 100 nM PD173074 and 10 U/mL heparin for 16 h. Relative caspase-3/7 activity was measured using ApoLive-Glo Multiplex Assay. Graphs show means ± SD from three independent experiments. Statistical significance: ***p < 0.001. The efficiency of p53 depletion was assessed using anti-p53 antibody. Anti-gamma-tubulin antibody served as a loading control. **d** Rescue effect of transient p53 expression on anti-apoptotic FGF1 activity in p53-negative G292 cells. Serum-starved G292 cells transiently transfected with p53_pcDNA-DEST47 (p53 transfected) or control pcDNA-DEST47 (–) vectors were stimulated with 200 ng/mL FGF1 in the presence of 100 nM PD173074 and 10 U/mL heparin for 16 h. Relative caspase-3/7 activity was measured using ApoLive-Glo Multiplex Assay. Graphs show means ± SD from three independent experiments. Statistical significance: **p < 0.01. The efficiency of p53 transfection was verified using an anti-p53 antibody. Anti-gamma-tubulin antibody served as a loading control. **e** Effect of p53 knockdown in U2OS cells stably transfected with FGF1. U2OS cells stably transfected with myc-FGF1_pcDNA3.1 (FGF1) or empty pcDNA3.1 (–) vectors were transfected with p53 siRNA or a non-targeting siRNA control (scr) and then treated with 5 μM actinomycin D in the presence of 100 nM PD173074 for 48 h. Cell viability was measured using Presto Blue reagent. Graphs show means ± SD from three independent experiments. Statistical significance: **p < 0.01. **f** PARP-1/2 cleavage in U2OS cells stably expressing FGF1 upon p53 knockdown. U2OS cells stably transfected with myc-FGF1_pcDNA3.1 (FGF1) or empty pcDNA3.1 (–) vectors were transfected with p53 siRNA or a non-targeting siRNA control (scr) and then treated with 5 μM actinomycin D in the presence of 100 nM PD173074 for 48 h. Anti-gamma-tubulin antibody served as a loading control. **g** Anti-apoptotic effect of FGF1 transient transfection in G292 cells expressing WT p53. G292 cells transiently transfected with a combination of p53_pcDNA-DEST47 (p53 transfected) or control pcDNA-DEST47 (–) vectors and myc-FGF1_pcDNA3.1 (FGF1) or empty pcDNA3.1 (–) vectors were treated with 50 μg/mL etoposide in the presence of 100 nM PD173074 for 48 h. Cell viability was measured using Presto Blue reagent. Graphs show means ± SD from three independent experiments. Statistical significance: *p < 0.05

Previously, we showed that translocated FGF1 and FGF2 protect the cell from apoptosis independently of FGF receptor activation [18]. In that study, we used p53-positive cell lines: mouse NIH 3T3 and human BJ fibroblasts, as well as human U2OS osteosarcoma cells stably transfected with FGFR1 (U2OS_R1) [19]. To test whether exogenously added FGF1 (rFGF1) reveals intracellular anti-apoptotic activity in p53-negative cell line naturally expressing FGFR1, we employed G292 cells. After induction of apoptosis by 24-h serum starvation, we monitored caspase 3/7 activity in U2OS_R1 and G292 cells 16 h after administration of recombinant FGF1 (200 ng/ml). In the absence of FGFR inhibitor, we observed a decrease in relative caspase-3/7 activity in G292 cells treated with exogenous FGF1 resulting from receptor activation and downstream signaling (Fig. S7). However, in the presence of PD173074, when FGFR-dependent signaling was blocked, the protective effect of FGF1 was only observed in U2OS_R1 cells (Fig. 6a). We confirmed FGF1 translocation in both cell lines by subcellular fractionation. U2OS_R1 and G292 cells were starved for 24 h and then treated with 100 ng/ml recombinant FGF1 for 6 h in the presence of 10 U/ml heparin and 100 nM PD173074. We observed FGF1 in cytosolic and nuclear fractions in both U2O_-R1 and G292 cells, confirming efficient FGF1 translocation in these cell lines (Fig. 6b). Treatment of cells in the presence of 10 nM Bafilomycin A1 (BafA1), an inhibitor of vacuolar proton pumps and consequently an inhibitor of FGF1 translocation, served as a negative control.

We then analyzed caspase 3/7 activity in U2OS_R1 cells in which p53 was knocked-down using a specific siRNA (Fig. 6c). 48 h after p53 siRNA transfection, U2OS_R1 cells were starved for 24 h and then treated with recombinant FGF1 in the presence of PD173074, as described above. Analysis of caspase 3/7 activity revealed that the protective effect of intracellular FGF1 was no longer observed in p53-depleted cells compared to cells transfected with non-targeting siRNA (scr siRNA) (Fig. 6c). Next, we wanted to verify whether it was possible to sensitize p53-negative cells to the protective effect of translocated FGF1. To this end, we transiently transfected G292 cells with wild type p53, induced apoptosis under serum starvation conditions and treated them with recombinant FGF1 in the presence of an FGFR kinase inhibitor. Consistent with the experiment shown in Fig. 6a, 16-h FGF1-treatment did not protect G292 cells from starvation-induced apoptosis (Fig. 6d). However, in G292 cells transfected with p53, we observed anti-apoptotic activity of translocated FGF1, as caspase 3/7 activity was significantly reduced (Fig. 6d). These results further support our hypothesis that the intracellular anti-apoptotic activity of FGF1 depends on the presence of p53.

We next examined the effect of p53 silencing on the anti-apoptotic activity of intracellular FGF1 in U2OS cells stably expressing FGF1. FGF1_U2OS and control U2OS cells were transfected with p53-targeting siRNA or scr siRNA and treated with 5 μM actinomycin D to induce apoptosis in the presence of 100 nM PD173074 for 48 h. Viability measurements showed that after p53 silencing, the anti-apoptotic activity of intracellular FGF1 was no longer observed (Fig. 6e). In addition, we monitored the progression of apoptosis by detecting PARP processing with western blot. As expected, p53 depletion led to increased PARP cleavage in FGF1_U2OS cells exhibiting the same level of cleaved PARP as in control U2OS cells (Fig. 6f), confirming previous results that p53 is crucial for the intracellular anti-apoptotic activity of FGF1.

Finally, we transiently transfected G292 cells with myc-FGF1 and wild-type p53. 48 h after transfection, G292 cells were treated with 50 μg/mL etoposide for 48 h to induce apoptosis in the presence of 100 nM PD173074, and then cell viability was measured. G292 cells transfected with both FGF1 and p53, showed significantly higher viability than cells transfected with FGF1 or p53 alone (Fig. 6g). Again, the protective effect of FGF1 could be restored by p53 expression.

### FGF1 interacts with p53 in cells

**Fig. 7:**
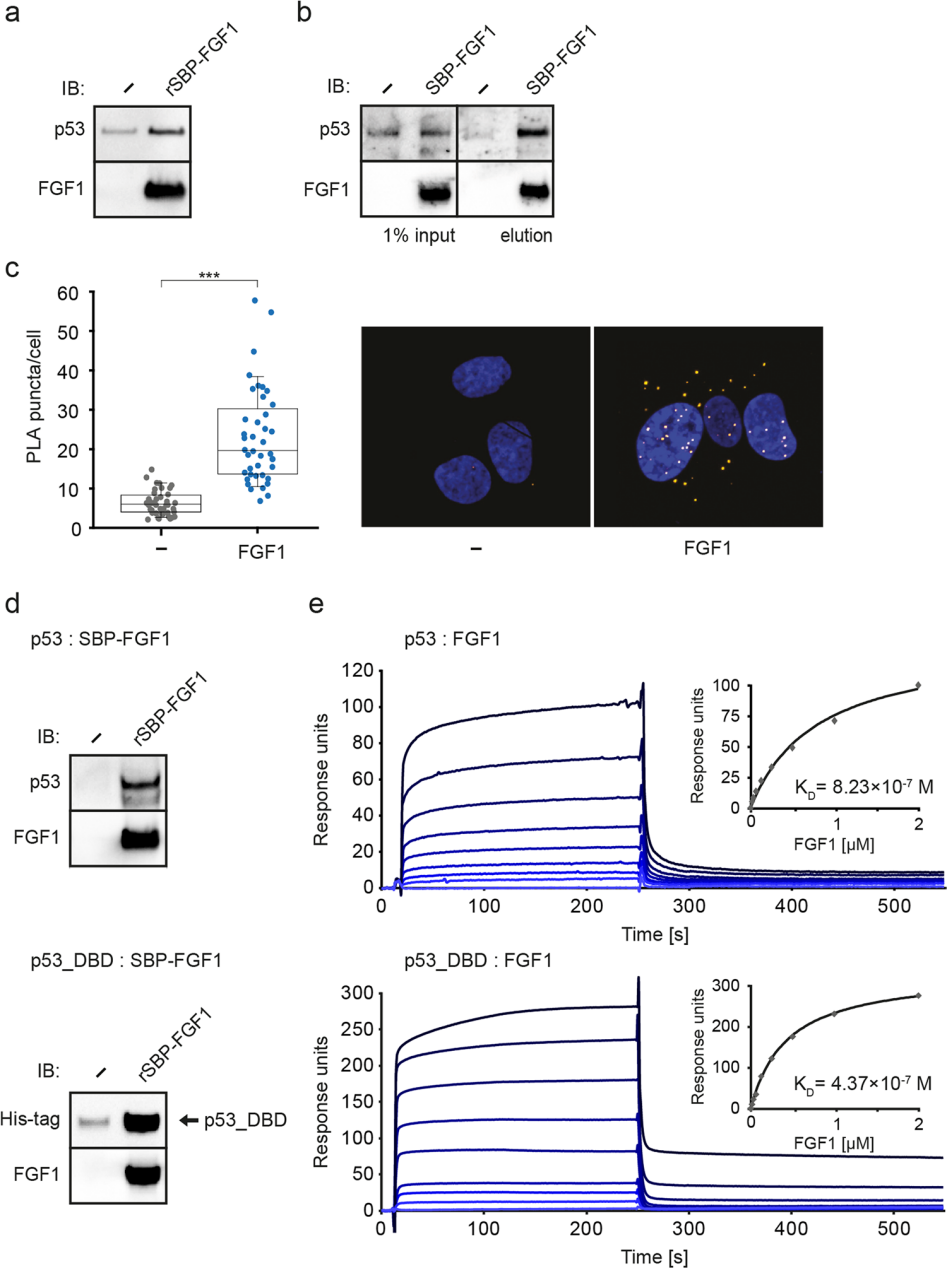
FGF1 interacts directly with p53. **a** Western blot analysis of pull-down experiment using U2OS cell lysate and recombinant SBP-FGF1. Cell lysates were incubated with SBP-FGF1 immobilized on Streptavidin-Agarose resin or with Streptavidin-Agarose resin alone for 1 h, then the resins were washed and proteins in the complex were analyzed using anti-p53 and anti-FGF1 antibodies. **b** Western blot analysis of pull-down experiment using U2OS stably transfected with SBP-FGF1_pcDNA3.1 (SBP-FGF1) or empty pcDNA3.1 (–) vectors. Cell lysates were incubated with Streptavidin-Agarose resin alone for 1 h, then the resins were washed and proteins in the complex were analyzed using anti-p53 and anti-FGF1 antibodies. **c** Proximity ligation assay in U2OS cells stably transfected with myc-FGF1_pcDNA3.1 (FGF1) or empty pcDNA3.1 (–) vectors showing the complexes of FGF1 and endogenous p53. Cells were fixed with 4% paraformaldehyde and subjected to the in situ PLA procedure using goat anti-FGF1 and mouse anti-p53 antibodies. A confocal z-stack including a whole cell was performed to observe the maximum amount of PLA signals. Cell nuclei were counterstained with DAPI. Representative images and quantification of PLA puncta per nucleus are shown. 41 images of each sample were analyzed. The box-and-whiskers graphs show the median, the 25th and 75th percentiles (box), and the 90th and 10th percentiles (whiskers). Statistical significance: ***p < 0.001. **d** FGF1 binds to p53 within its DNA-binding domain. Western blot analysis of pull-down experiment using recombinant His-tagged full-length p53 and its DNA-binding domain (p53_DBD). p53 proteins were incubated with recombinant SBP-FGF1 immobilized on Streptavidin-Agarose resin or Streptavidin-Agarose resin alone for 1 h, then the resins were washed and proteins in the complex were analyzed using anti-His-tag and anti-FGF1 antibodies. **e** Kinetics of p53: FGF1 and p53_DBD: FGF1 interaction assessed with SPR. The FGF1 protein at the concentrations from 0.016 μM to 2.048 μM was injected on CM5 sensor surface with p53 or DNA-binding domain of p53 immobilized at 1000 RU or 800 RU, respectively. Equilibrium dissociation constant (K_D_) was calculated from saturation binding curve

Previously, our group identified p53 as a novel FGF1 binding partner [39]. Here, we verified p53-FGF1 interaction in U2OS cells. We first performed a pull-down experiment using recombinant SBP-FGF1 (rSBP-FGF1) immobilized on Streptavidin-Agarose resin and U2OS cell lysate. Western blot analysis confirmed the interaction of FGF1 with p53 from U2OS cell lysate, as p53 was detected in the sample containing rSBP-FGF1 and absent in the negative control (Streptavidin-Agarose resin alone) (Fig. 7a). Next, to verify whether FGF1 interacts with p53 in cells, we used U2OS cells stably transfected with SBP-FGF1_pcDNA3.1 vector (SBP-FGF1_U2OS) or U2OS control cells (transfected with empty pcDNA3.1 vector) and performed a pull-down experiment in a manner similar to that described above. Western blot analysis confirmed that FGF1 forms a complex with p53 in U2OS cells (Fig. 7b).

Then, to visualize and further characterize this interaction in cells, we applied the in situ Proximity Ligation Assay in U2OS cells stably expressing myc-FGF1 (FGF1_U2OS). For this purpose, FGF1_U2OS and U2OS control cells (a negative control) were incubated with mouse anti-p53 and goat anti-FGF1 antibodies. Additional controls were also performed for anti-p53 and anti-FGF1 antibodies. As shown in Fig. 7c, we confirmed the p53-FGF1 interaction in FGF1_U2OS cells. The pattern of p53-FGF1 interaction is evenly distributed in the cell, suggesting a cytoplasmic localization of p53-FGF1 complexes.

### FGF1 interacts directly with p53 DNA-binding domain

To gain further insight into the FGF1-p53 interaction, we produced His-p53 (1-393) and His-DBD (94-312) in a bacterial expression system (Fig. S8). Recombinant full-length p53 and its DNA-binding domain (DBD) were then used in a pull-down experiment with recombinant SBP-FGF1 immobilized on Streptavidin-Agarose resin. Protein complexes eluted from the beads were analyzed by western blotting, revealing that SBP-FGF1 binds both full-length p53 and p53 DBD proteins (Fig. 7d).

To confirm the direct interaction between FGF1 and the DNA-binding domain of p53 and to determine the binding parameters, we used surface plasmon resonance (SPR) technique. Samples of recombinant FGF1 at different concentrations were injected onto a CM5 sensor chip with immobilized p53 or p53_DBD. The shape of the interaction curves confirmed that FGF1 binds to full-length p53, as well as to the DBD fragment of p53 (Fig. 7e). Using the fitted saturation binding curve derived from the equilibrium binding response plotted against the concentrations of FGF1, we determined the K_D_ for the interaction of FGF1 with p53 and p53_DBD as 5.59 × 10^–7^ M and 4.37 × 10^–7^ M, respectively.

## Discussion

FGF1 is well characterized in the context of its extracellular activity, which includes the binding and activation of FGF receptors, resulting in the triggering of signaling cascades leading to cell proliferation and increased survival. In parallel, FGF1 is translocated into the cell by endocytosis in complex with the receptor [17]. Internalized FGF1 interacts with many intracellular proteins [40]. Some of these are proteins critical for its trafficking from endosomes to the cytosol and then to the nucleus, such as Hsp90 or LRRC59 [41, 42]. However, a large fraction of the proteins we identified as partners of FGF1 are proteins involved in apoptosis-related processes, including nucleophosmin, gelsolin and p53 [39]. We previously showed that the role of translocated FGF1 is to inhibit apoptosis, independent of FGFR stimulation [18]. In the current study, we have focused on describing the role of FGF1-p53 interactions for the intracellular pro-survival activity of FGF1.

The relationship between FGF1 and p53 was first described by Boulau et al. [43]. They found that p53 activation induces FGF1 downregulation and showed that intracellular FGF1 inhibits p53-dependent apoptosis in rat embryonic fibroblast and PC12 cells [43, 44]. The same group suggested that FGF1-generated cellular protection is regulated by its phosphorylation [45, 46]. They also found that FGF1 overexpression in ovarian granulosa cancer cells induced chemotherapy resistance and p53 accumulation in mitochondria [47].

Here, we demonstrate that the direct interaction of FGF1 with p53 is responsible for the anti-apoptotic effect of intracellular FGF1. We first showed that FGF1 expression (transient or stable) made U2OS cells more resistant to various stress stimuli, such as staurosporine, anisomycin and actinomycin D, even when FGFR signaling was completely blocked. Intracellular FGF1 increased viability and inhibited caspase activity, reducing the number of apoptotic cells. To gain more insight into the mechanism of the anti-apoptotic activity of FGF1 inside the cell, we induced apoptosis in U2OS cells stably transfected with FGF1 through both intrinsic and extrinsic pathways. We showed that intracellular FGF1 could not protect U2OS cells in which apoptosis was induced via the extrinsic pathway using the Apo2/TRAIL protein [33]. We used staurosporine, anisomycin and actinomycin D as inducers of the intrinsic pathway, but also compounds that specifically activate p53, such as etoposide, doxorubicin and camptothecin [26–32]. For each of these, we observed significantly higher viability of U2OS cells expressing FGF1 than control cells. In a further step, we examined the effect of FGF1 on ER stress using brefeldin A and thapsigargin [34–36]. Again, we did not observe any protection from FGF1 against apoptosis induced in this way.

Then, using specific compounds or transfection with a construct encoding the Bax protein, we induced apoptosis at the permeabilization stage of the mitochondrial membrane [22, 37, 38] in U2OS cells or U2OS cells stably expressing FGF1. Our results suggest that FGF1 does not inhibit Bax insertion into the mitochondrial membrane, as we did not observe any anti-apoptotic effect of FGF1. Therefore, we suggest that the anti-apoptotic activity of intracellular FGF1 is related to events preceding permeabilization of the outer mitochondrial membrane.

Previously, a protective effect of FGF1 has been reported for rat embryonic fibroblast, PC12 and COV434 cell lines [43, 44, 47]. We observed the anti-apoptotic activity of intracellular (ectopically expressed) FGF1 in several other cell types, including human BJ fibroblasts, osteosarcoma cells stably transfected with FGFR1 (U2OS_R1 [19]), breast cancer cell line MCF7 and human embryonic kidney 293 (HEK 293) cells, suggesting that the observed effect is a common phenomenon. All of the cell lines we studied naturally expressed WT p53, so we decided to test the anti-apoptotic properties of intracellular FGF1 also in p53-null cells. We performed experiments using G292 osteosarcoma and PC3 prostate cancer cell lines that do not express the p53 protein [48–51]. In these cells, we did not observe a protective effect of FGF1 expression against apoptosis. These results strongly suggest that a functional p53 protein is crucial for the anti-apoptotic properties of intracellular FGF1.

In the next step, we treated with exogenous FGF1 two osteosarcoma lines expressing FGFR1 (G292 expressing endogenous FGFR1 [52] and U2OS stably transfected with FGFR1 (U2OS_R1)), but differing in p53 status. Both of these lines were able to translocate FGF1, but with blocked receptor kinase activity, only in U2OS_R1 cells carrying the wild-type form of p53 did we observe a protective effect of translocated FGF1. Furthermore, when U2OS cells stably transfected with FGF1 were silenced with p53, we found that these cells behaved like G292 cells and were no longer protected from apoptosis by translocated FGF1. In contrast, transfection of G292 cells with wild-type p53 resulted in an anti-apoptotic effect of translocated and ectopically expressed FGF1. We subsequently examined the knock-down effect of p53 on the anti-apoptotic activity of intracellular FGF1 in U2OS cells stably expressing FGF1 (FGF1_U2OS). When p53 was silenced, the pro-survival activity of intracellular FGF1 was no longer observed, confirming previous results that p53 is critical for the intracellular anti-apoptotic activity of FGF1.

In our previous work, using recombinant proteins and cell lysates, we showed that the binding of FGF1 to p53 is a direct interaction [39]. Here, these data were confirmed by showing for the first time a direct complex between the two proteins in cells using a proximity ligation assay. These results allow us to hypothesize that the anti-apoptotic activity of intracellular FGF1 is strongly linked to its interaction with p53. To better characterize the FGF1-p53 interaction, we produced the p53 protein and its DNA-binding domain (p53 DBD) in a bacterial system. Tomita et al*.* showed that the p53_DBD, in addition to its importance for p53 transcriptional activity, is also involved in the p53-Bcl-2/Bcl-XL interaction leading to Bcl-2/Bcl-XL inhibition enabling subsequent mitochondrial permeabilization [53]. By analyzing interactions between recombinant proteins, we found that the DBD of p53 is involved in FGF1 binding. This interaction is relatively strong, as the K_D_ is submicromolar and is close to the K_D_ obtained for the full-length protein.

FGF1 has been reported to regulate the transcriptional activity of p53 in etoposide-treated PC12 cells by reducing the mRNA levels of PUMA, p21 and Bax[44]. However, a recent study by Manousakidi et al*.* shows that FGF1 regulates the mitochondrial localization of p53 in response to etoposide treatment in COV434 cells, without affecting its transcriptional activity [47]. Our results, which indicate a role for the DNA-binding domain of p53, raise the possibility that FGF1 may regulate both transcription-dependent and transcription-independent p53 activity. It is possible that FGF1 binding to p53 via the DBD may block the interaction of both p53 with DNA and with Bcl-2 family proteins, thereby affecting two different modes of apoptotic p53 activity.

Here we have shown that FGF1 interacts directly with p53 via DBD binding and thus inhibits apoptosis in various p53-positive cell types by the intrinsic pathway. The presence of functional p53 is critical for the pro-survival activity of FGF1. Nevertheless, further studies on the transcriptional activity of p53, its mitochondrial translocation and interactions with pro- and anti-apoptotic Bcl-2 family proteins are needed to better understand the mechanism of the intracellular anti-apoptotic activity of FGF1.

### Supplementary Information

Below is the link to the electronic supplementary material.

Supplementary file1 (PDF 685 KB)

## Data Availability

The datasets obtained during the current study are available upon request.
