# Supplementary material for: Intracellular FGF1 protects cells from apoptosis through direct interaction with p53
Source: Cell Mol Life Sci. 2023 Oct 2;80(10):311. doi: 10.1007/s00018-023-04964-9 (PMC10545594; doi:10.1007/s00018-023-04964-9)
Supplement: Supplementary file 1 — Supplementary file1 (PDF 685 KB) [file 18_2023_4964_MOESM1_ESM.pdf]

Supplementary material

Intracellular FGF1 protects cells from apoptosis through direct interaction with p53

Agata Lampart[1], Daniel Krowarsch[2], Martyna Biadun[1,2], Vigdis Sorensen[3,4], Jakub Szymczyk[1], Katarzyna Sluzalska[1], Antoni Wiedlocha[4,5], Jacek Otlewski[1], Malgorzata Zakrzewska[1]*

[1]Department of Protein Engineering, Faculty of Biotechnology, University of Wroclaw, Wroclaw, Poland

[2]Department of Protein Biotechnology, Faculty of Biotechnology, University of Wroclaw, Wroclaw, Poland

[3]Advanced Light Microscopy Core Facility, Dept. Core Facilities, Institute for Cancer Research, The Norwegian Radium Hospital, Oslo University Hospital, Montebello, Oslo, Norway

[4]Centre for Cancer Cell Reprogramming, Institute of Clinical Medicine, Faculty of Medicine, University of Oslo, Montebello, Oslo, Norway

[5]Department of Molecular Cell Biology, Institute for Cancer Research, Oslo University Hospital, Montebello, Oslo, Norway

* Corresponding author, e-mail: malgorzata.zakrzewska@uwr.edu.pl

Supplementary figure legends

Fig. S1 (a) Viability of U2OS cells transiently transfected with myc-FGF1_pcDNA3.1 (FGF1) or control pcDNA3.1 (-) vectors and treated with 10 μM anisomycin or 5 μM actinomycin D in the absence or presence of FGFR kinase inhibitors (10 nM BGJ398 or 1 μM ARQ087) for 24 h. Cell viability was measured using Presto Blue reagent. Graphs show means ± SD from three independent experiments. Statistical significance: *p<0.05, **p<0.01. **(b)** Western blot analysis of FGFR-dependent signaling in U2OS cells transiently transfected with myc-FGF1_pcDNA3.1 (FGF1) or control pcDNA3.1 (-) vectors and treated with 1 μM staurosporine in the presence of 100 nM PD173074 or 10 nM BGJ398 for 6 h and 24 h. FGFR downstream signaling was assessed using an anti-phospho-ERK1/2 antibody. Anti-γ-tubulin antibody was used as a loading control

Fig. S2 (a) Viability of U2OS cells stably transfected with myc-FGF1_pcDNA3.1 (FGF1) or empty pcDNA3.1 (-) vectors (two clones each, #1, #2) treated with 1 μM staurosporine, 10 μM anisomycin or 5 μM actinomycin D. Cell viability was measured using Presto Blue reagent 24 h after treatment. Graphs show means ± SD from three independent experiments. Statistical significance: *p<0.05, **p<0.01. **(b)** Viability of U2OS cells stably transfected with myc-FGF1_pcDNA3.1 (FGF1) or empty pcDNA3.1 (-) vectors (two clones each, #1, #2) treated with 5 μM actinomycin D in the presence of FGFR kinase inhibitor (100 nM PD173074). Cell viability was measured using Presto Blue reagent 48 h after treatment. Graphs show means ± SD from three independent experiments. Statistical significance: *p<0.05, **p<0.01. **(c)** Western blot analysis of FGFR-dependent signaling in U2OS cells stably transfected with myc-FGF1_pcDNA3.1 (FGF1) vector and treated with 1 μM staurosporine, 10 μM anisomycin or 5 μM actinomycin D in the presence of 100 nM PD173074 for 15 min, 6 h and 24 h. FGFR downstream signaling was assessed using an anti-phospho-ERK1/2 antibody. Anti-gamma-tubulin antibody was used as a loading control.

(d) Viability of U2OS cells stably transfected with myc-FGF1_pcDNA3.1 (FGF1) or empty pcDNA3.1 (-) vectors treated with 1 μM staurosporine, 10 μM anisomycin or 5 μM actinomycin D in the presence of FGFR kinase inhibitors (10 nM BGJ398 or 1 μM ARQ087). Cell viability was measured using Presto Blue reagent 24 h after treatment. Graphs show means ± SD from three independent experiments. Statistical significance: *p<0.05, **p<0.01. **(e)** Relative caspase-3/7 activity measured in U2OS cells stably transfected with myc-FGF1_pcDNA3.1 (FGF1) or empty pcDNA3.1 (-) vectors after 24-h treatment with 1 μM staurosporine, 10 μM anisomycin or 5 μM actinomycin D in the absence or presence of 10 nM BGJ398 using ApoLive-Glo Multiplex Assay. Graphs show means ± SD of three independent experiments. Statistical significance: *p<0.05, **p<0.01

Fig. S3 (a) Viability of U2OS cells stably transfected with myc-FGF1_pcDNA3.1 (FGF1) or empty pcDNA3.1 (-) vectors treated with 50 μg/mL etoposide in the absence or presence of FGFR kinase inhibitors (10 nM BGJ398 or 1 μM ARQ087). Cell viability was measured using Presto Blue reagent 24 h after treatment. Graphs show means ± SD from three independent experiments. Statistical significance: *p<0.05, **p<0.01. **(b)** Western blot analysis of FGFR-dependent signaling in U2OS cells stably transfected with myc-FGF1_pcDNA.3.1 (FGF1) vector and treated with 50 μg/mL etoposide in the presence of 100 nM PD173074 for 15 min, 6 h and 24 h. FGFR downstream signaling was assessed using an anti-phospho-ERK1/2 antibody. Anti-gamma-tubulin antibody was used as a loading control. **(c)** Relative caspase-3/7 activity measured in U2OS cells stably transfected with myc-FGF1_pcDNA3.1 (FGF1) or empty pcDNA3.1 (-) vectors after 24-h treatment with 50 μg/mL etoposide in the absence or presence of 100 nM PD173074 or 10 nM BGJ398 using ApoLive-Glo Multiplex Assay. Graphs show means ± SD of three independent experiments. Statistical significance: *p<0.05, **p<0.01

Fig. S4 Viability of U2OS cells stably transfected with myc-FGF1_pcDNA.3.1 (FGF1) or empty pcDNA.3.1 (-) vectors treated with 300 ng/mL Fas ligand in the presence of FGFR kinase inhibitor (100 nM PD173074). Cell viability was measured using Presto Blue reagent 24 h after treatment. Graphs show means ± SD from three independent experiments. No statistical significance

Fig. S5 Viability of U2OS cells stably transfected with myc-FGF1_pcDNA.3.1 (FGF1) or empty pcDNA.3.1 (-) vectors treated with 30 μM BTSA1 in the presence of FGFR kinase inhibitor (100 nM PD173074). Cell viability was measured using Presto Blue reagent 24 h after treatment. The graph shows means ± SD from three independent experiments. No statistical significance (left panel). PARP-1/2 cleavage determined in U2OS cells stably transfected with myc-FGF1_pcDNA.3.1 (FGF1) or empty pcDNA.3.1 (-) vectors after 24-h treatment with 30 μM BTSA1 in the presence of 100 nM PD173074. Anti-gamma-tubulin antibody served as a loading control (right panel)

Fig. S6 Western blot analysis of FGF1 protein levels in p53-positive (MCF7, BJ and HEK 293) and p53-negative (G292 and PC3) cell lines 48 h after transient transfection with myc-FGF1_pcDNA.3.1 (FGF1) or empty pcDNA.3.1 (-) vectors. Anti-gamma-tubulin antibody served as a loading control

Fig. S7 Relative caspase-3/7 activity measured using ApoLive-Glo Multiplex Assay in serum-starved G292 cells stimulated with 200 ng/mL FGF1 in the presence of 10 U/mL heparin for 16 h. Graphs show means ± SD of three independent experiments. Statistical significance: ***$p<0.001$

Fig. S8 SDS-PASGE analysis of expression and purification of recombinant His-tagged full length p53 (left panel) and p53 DBD (right panel). Both proteins were produced in *E.Coli* Arctic Express strain. Full-length his-p53 was purified by affinity chromatography using

Ni-NTA column followed by ion exchange chromatography (IEX). His-tagged p53_DBD was purified using Ni-NTA column and dialysis

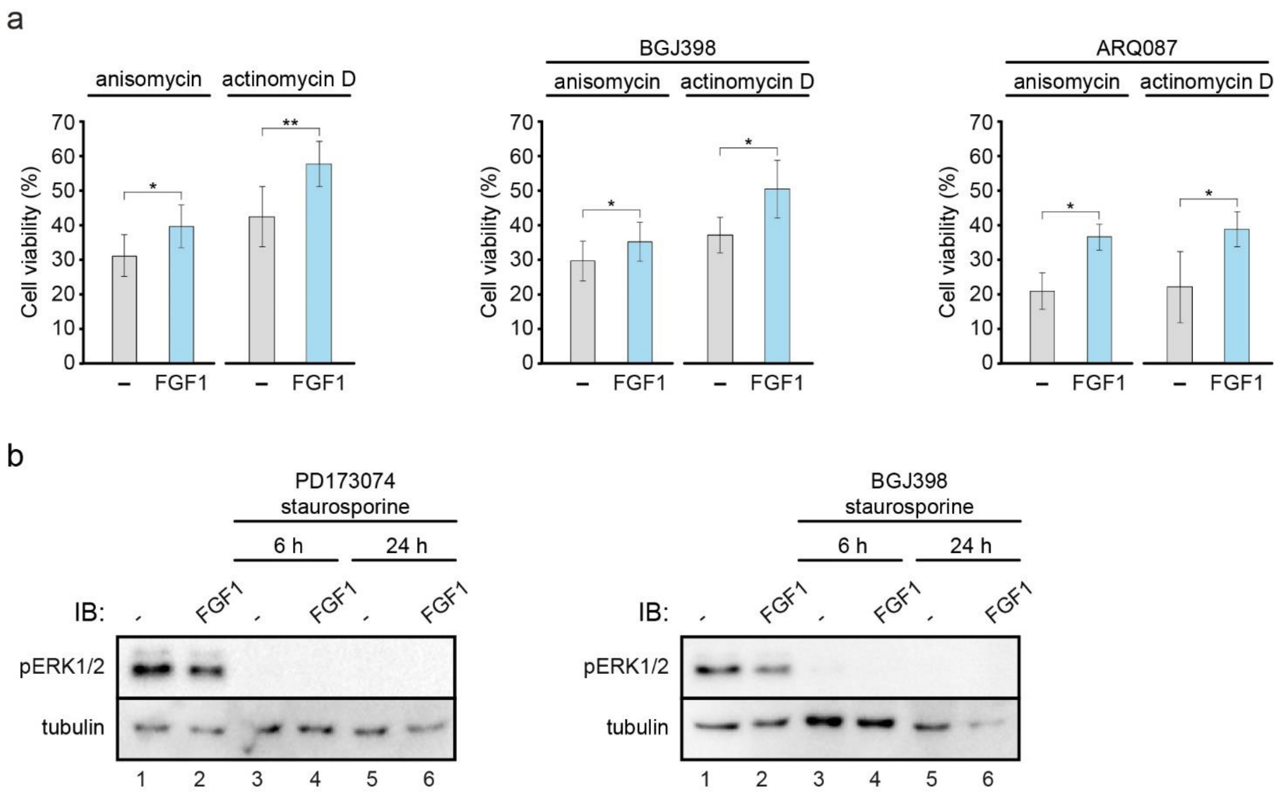
a
anisomycin
actinomycin D
BGJ398
ARQ087
Cell viability (%)
FGF1
b
PD173074
staurosporine
BGJ398
6 h
24 h
IB:
pERK1/2
tubulin

Fig. S1

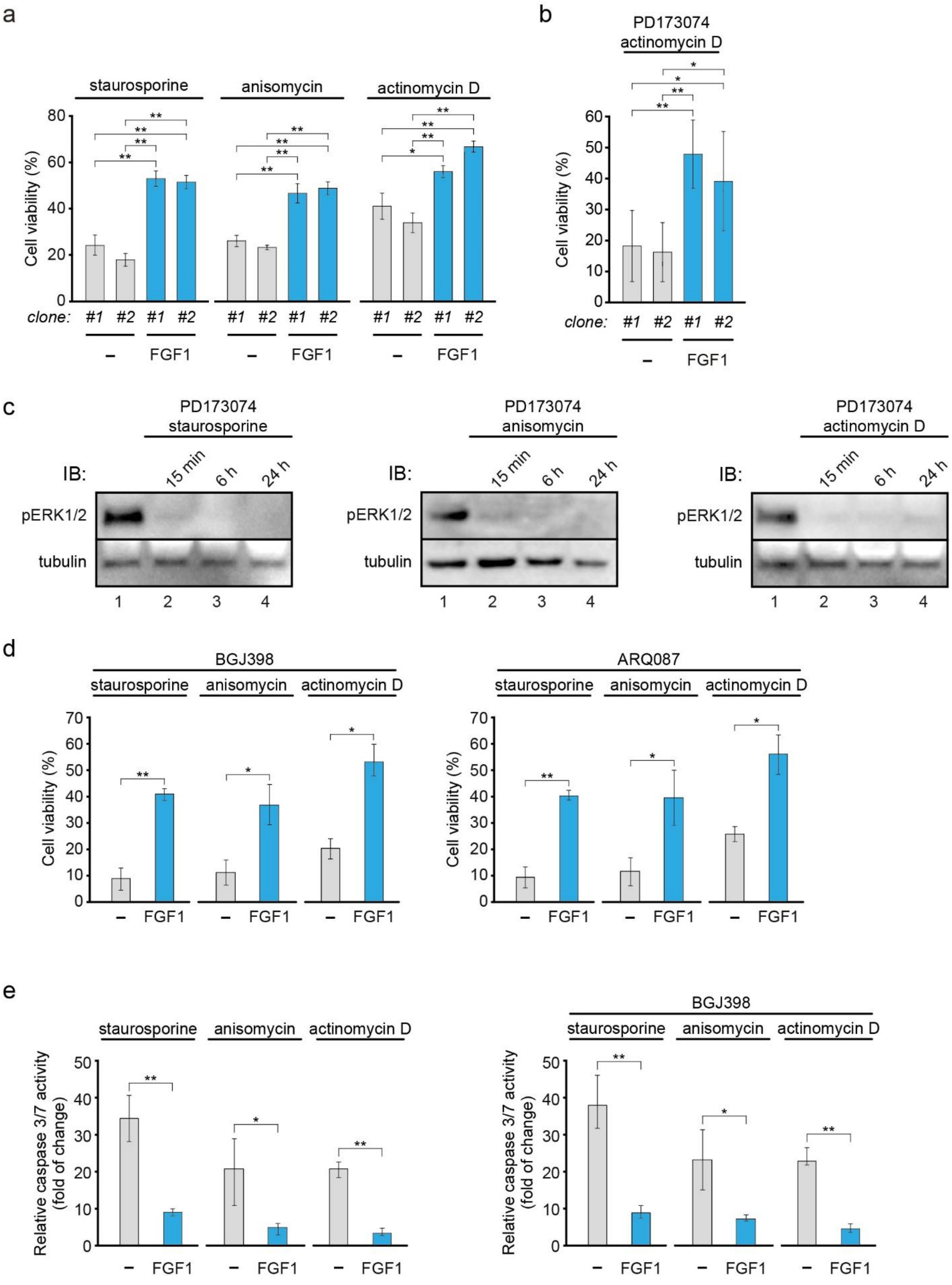
a
staurosporine
anisomycin
actinomycin D
Cell viability (%)
clone: #1 #2 #1 #2
–
FGF1
b
PD173074
actinomycin D
c
PD173074
staurosporine
anisomycin
actinomycin D
IB:
15 min
6 h
24 h
pERK1/2
tubulin
1 2 3 4
d
BGJ398
ARQ087
e
Relative caspase 3/7 activity (fold of change)

Fig. S2

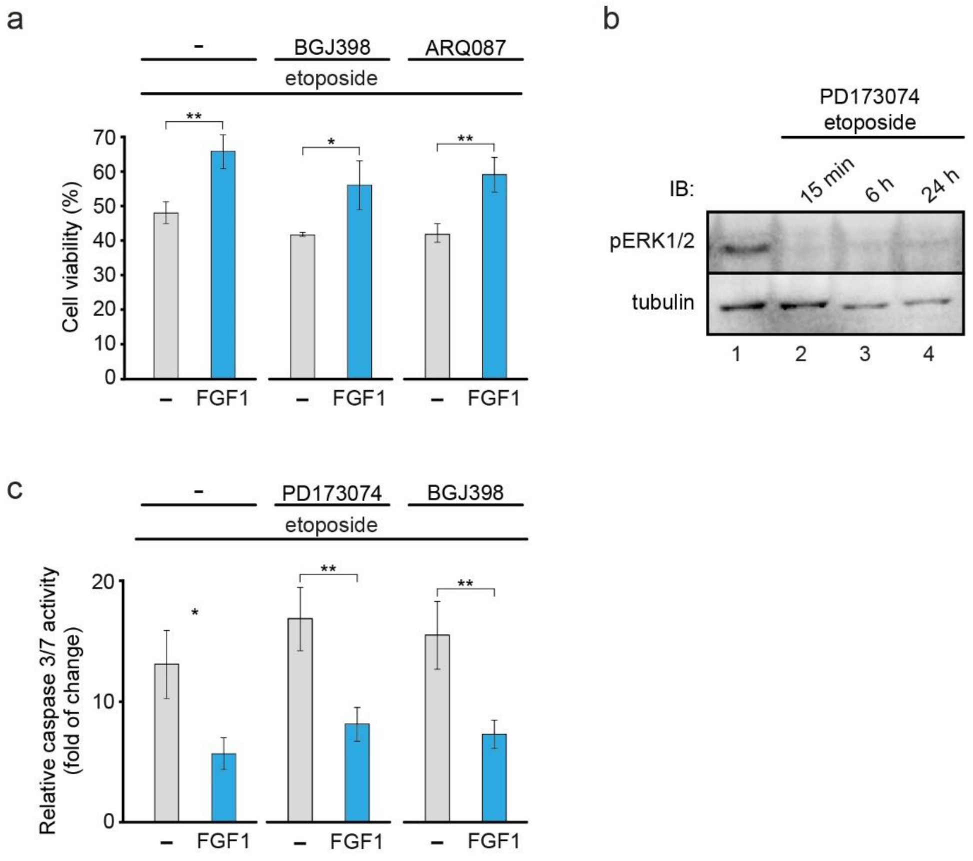

a
-
BGJ398
ARQ087
etoposide
**
*
**
70
60
50
40
30
20
10
0
Cell viability (%)
–
FGF1
–
FGF1
–
FGF1
b
PD173074
etoposide
IB:
15 min
6 h
24 h
pERK1/2
tubulin
1
2
3
4
c
-
PD173074
BGJ398
etoposide
20
10
0
*
**
**
Relative caspase 3/7 activity (fold of change)
–
FGF1
–
FGF1
–
FGF1

Fig S3

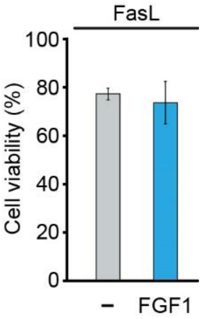

Fig. S4

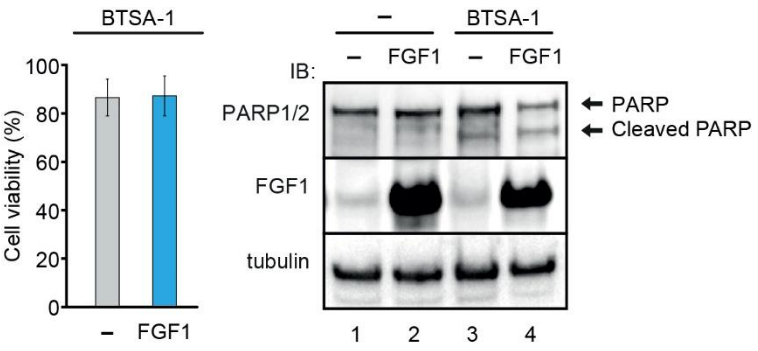

BTSA-1
Cell viability (%)
100
80
60
40
20
0
–
FGF1
–
BTSA-1
–
FGF1
–
FGF1
IB:
PARP1/2
FGF1
tubulin
PARP
Cleaved PARP
1
2
3
4

Fig. S5

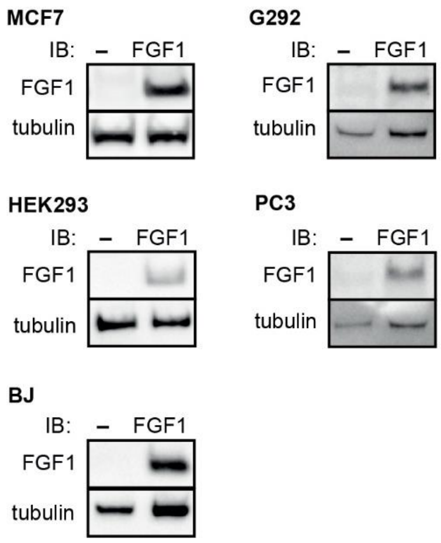
MCF7
IB: – FGF1
FGF1
tubulin
G292
IB: – FGF1
FGF1
tubulin
HEK293
IB: – FGF1
FGF1
tubulin
PC3
IB: – FGF1
FGF1
tubulin
BJ
IB: – FGF1
FGF1
tubulin

Fig. S6

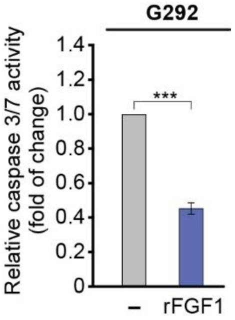
G292
Relative caspase 3/7 activity
(fold of change)
1.4
1.2
1.0
0.8
0.6
0.4
0.2
0
***
–
rFGF1

Fig. S7

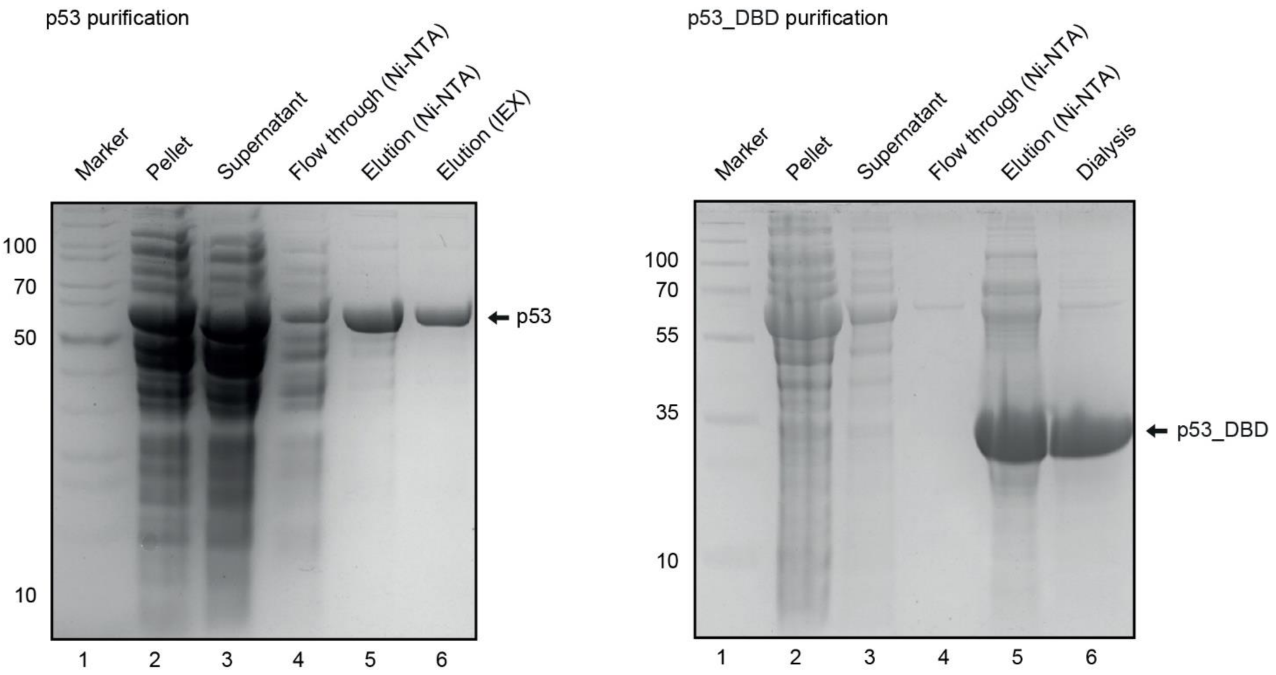
p53 purification
Marker
Pellet
Supernatant
Flow through (Ni-NTA)
Elution (Ni-NTA)
Elution (IEX)
100
70
50
10
p53
1
2
3
4
5
6
p53_DBD purification
Marker
Pellet
Supernatant
Flow through (Ni-NTA)
Elution (Ni-NTA)
Dialysis
100
70
55
35
10
p53_DBD
1
2
3
4
5
6

Fig. S8
